# Effects of patient factors on noninvasive liver stiffness measurement using acoustic radiation force impulse elastography in patients with chronic hepatitis C

**DOI:** 10.1186/1471-230X-12-105

**Published:** 2012-08-10

**Authors:** Sheng-Hung Chen, Yu-Fen Li, Hsueh-Chou Lai, Jung-Ta Kao, Cheng-Yuan Peng, Po-Heng Chuang, Wen-Pang Su, I-Ping Chiang

**Affiliations:** 1 Division of Hepatogastroenterology, Department of Internal Medicine, China Medical University Hospital, No 2 Yuh-Der Road, Taichung, 40402, Taiwan; 2 China Medical University, Taichung, 40402, Taiwan; 3 Institute of Biostatistics Center, China Medical University, Taichung, 40402, Taiwan; 4 College of Chinese Medicine, China Medical University, Taichung, 40402, Taiwan; 5 School of Medicine, China Medical University, Taichung, 40402, Taiwan; 6 Department of Pathology, China Medical University Hospital, Taichung, 40402, Taiwan

**Keywords:** Liver fibrosis, Cirrhosis, Acoustic radiation force impulse, FibroTest, ActiTest, Chronic hepatitis C

## Abstract

**Background:**

Previous research has shown variation in the effects of patient factors, including hepatic necroinflammatory activity, on liver stiffness measurement (LSM). This prospective study attempts to identify explanatory factors for LSM in patients with chronic hepatitis C (CHC) using acoustic radiation force impulse (ARFI) technology.

**Methods:**

A cohort of 127 Taiwanese patients with CHC underwent ARFI LSM and immediate percutaneous liver biopsy. This study compares the concurrent diagnostic performances of LSM and FibroTest using receiver operating characteristic (ROC) curves. Three multiple linear regression models were used to evaluate the significance of concurrent patient factors in explaining LSM.

**Results:**

To classify METAVIR fibrosis (F) stages, the areas under ROC curves (AUCs) were ARFI LSM, 0.847 (95% confidence interval (CI), 0.779-0.914) and FibroTest, 0.823 (95% CI, 0.748-0.898), for F1 versus F2-4; ARFI LSM, 0.902 (95% CI, 0.835-0.970) and FibroTest, 0.812 (95% CI, 0.735-0.888), for F1-2 versus F3-4; ARFI LSM, 0.831 (95% CI, 0.723-0.939) and FibroTest, 0.757 (95% CI, 0.648-0.865), for F1-3 versus F4. After adjusting for other demographic and biological covariates, biochemical and histological necroinflammatory factors consistently explained LSM. Factors included serum alanine aminotransferase (ALT)/upper limit of normal (ULN) categories (model R^2^ = 0.661, adjusted R^2^ = 0.629), ActiTest A scores (R^2^ = 0.662, adjusted R^2^ = 0.636), and METAVIR activity (A) grades (R^2^ = 0.651, adjusted R^2^ = 0.620). METAVIR F stages, body mass index, and platelet count were also independently associated with LSM. Necroinflammatory degrees, including ALT/ULN, ActiTest A scores, and METAVIR A grades, explained the false positivity of liver fibrosis staging using ARFI LSM.

**Conclusions:**

The degree of hepatic necroinflammatory activity independently and significantly exaggerated liver fibrosis evaluation using ARFI LSM. However, comparisons with concurrent FibroTest indicate that ARFI LSM may be a promising alternative, or adjunctive single indicator, for liver fibrosis evaluation in patients with CHC.

## Background

Hepatitis C virus (HCV) infection is a leading cause of cirrhosis and hepatocellular carcinoma (HCC) worldwide [1]. In the current era of antiviral and antifibrotic treatments, clinical and research demands are increasing significantly worldwide for noninvasive surveillance methods for liver fibrosis. These methods are necessary to evaluate the progression and regression of liver fibrosis [2-6].

Recent investigations have proposed liver stiffness measurement (LSM) using acoustic radiation force impulse (ARFI) elastography as a novel, reliable, and accurate noninvasive solution for evaluating liver fibrosis [7-15]. However, the cutoff variability in previous studies may be the result of a limited number of samples with diverse hepatitis etiologies and variable sample characteristics, including demographic and biological covariates, ethnicity, and prolonged intervals between liver biopsy and LSM. In several of these studies, the time intervals between biopsy and LSM were as long as 6 to 15 months [8,12,13]. These may have affected the LSM results because of potential histological progression and the dynamic fibrogenic process.

Although previous studies [7,8,10,12-15] have implemented concurrent FibroScan (Echosens, Paris, France) and ARFI, few studies [8,9] have compared [16] the diagnostic performances or discrimination capabilities of ARFI and FibroTest (BioPredictive, Paris, France) in evaluating liver fibrosis. Local LSM spectra using ARFI technology are still lacking for Taiwanese patients with chronic hepatitis C (CHC).

In addition to several factors related to the stiffness measurement itself [14,17-19], several patient factors, including hepatic necroinflammatory activity [12,13,15,20-30], cholestasis [31,32], and cardiac congestion [33], affect the LSM values in liver fibrosis evaluation. Therefore, independent significant explanatory patient factors should be evaluated comprehensively with caution when interpreting or fine-tuning the results of ARFI LSM. However, previous studies have shown variation in the effects of patient factors, including hepatic necroinflammatory activities, on LSM using FibroScan or ARFI [12,13,28,32]. Despite the wide use of hepatic elastography, few studies [12,29,30] have correlated ARFI LSM with concurrent histological steatosis and necroinflammatory categories, including the METAVIR activity (METAVIR A) grades, and not merely blood test results. Few studies have measured the necroinflammatory degrees using ActiTest (BioPredictive, Paris, France) [34].

To date, clinically applicable noninvasive tests do not focus on the exact staging of disease, but rather on dividing patients into categories of milder versus more significant fibrosis and cirrhosis [5]. The adjusted effects of METAVIR fibrosis (METAVIR F) stages on ARFI LSM, and the potential limitations of ARFI LSM (especially the false positivity in liver fibrosis staging) also have yet to be fully elucidated.

This prospective study examines the contributions of concurrent patient factors, and especially the histological covariates, to ARFI LSM. This study also examines the adjusted effects of METAVIR F stages on ARFI LSM, with the purpose of evaluating diagnostic performance using the simple and noninvasive single indicator ARFI LSM as compared with concurrent FibroTest. Finally, this study identifies the optimal real time ARFI LSM cutoff values for liver fibrosis staging in Taiwanese patients with CHC.

## Methods

### Patients

From November 2010 to October 2011, a cohort of 142 consecutive Taiwanese patients participated in this prospective, operator-blind study. The participants were referred to the liver center for percutaneous liver biopsy prior to the initiation of standard of care (SOC) for CHC. This study defines CHC as positive serum anti-HCV antibody (Abbott Laboratories, Abbott Park, Illinois, USA) for more than six months with the presence of serum HCV RNA (Cobas Amplicor HCV Monitor 2.0; Roche Diagnostics, Branchburg, New Jersey, USA).

Five patients refused, or were contraindicated for, percutaneous liver biopsy. Thus, a total of 137 patients underwent percutaneous liver biopsy within one hour of receiving blood tests (including those for FibroTest) and stiffness measurements, preceded by three hours of fasting [35].

Patients with any of the following conditions or history were excluded from the sample: interferon or nucleos(t)ide analogue treatment, exposure to hepatotoxic drugs or chemicals, primary biliary cirrhosis, primary sclerosing cholangitis, Wilson’s disease, autoimmune hepatitis, alcoholic liver disease (ALD), hepatitis B virus (HBV) coinfection, human immunodeficiency virus (HIV) coinfection, liver abscess, acute hepatitis, extrahepatic cholestasis [31,32], severe hemolysis, Gilbert's syndrome with high unconjugated hyperbilirubinemia, autoimmune disorders, myeloproliferative disorders, thalassemias, schistosomiasis, major abdominal surgery, cardiac congestion [33], blood product transfusion within the previous 30 days, pregnancy, liver cancer, serum creatinine higher than 221 umol/L (2.5 mg/dL), hepatic encephalopathy, refractory ascites [36], and variceal bleeding.

This study conforms to the Helsinki Declaration of 1975 and was approved by the Institutional Review Board of China Medical University Hospital (CMUH IRB No. DMR99-IRB-240). All individuals provided written informed consent prior to study enrollment.

### FibroTest and ActiTest

Serum markers including α2-macroglobulin, alanine aminotransferase (ALT), apolipoprotein A1, total bilirubin, γ-glutamyl transpeptidase (GGT) and haptoglobin were tested in the same laboratory, and results were then sent to http://www.biopredictive.com to determine a measure of liver fibrosis (FibroTest F score) and necroinflammatory activity (ActiTest A score) using patented artificial intelligence algorithms [37,38].

### ARFI LSM

ARFI technology [39] was integrated into a conventional ultrasound system (Acuson S2000 with a Siemens 4C1 curved array, 4.00 MHz for B-mode, 2.67 MHz for push pulses and 3.08 MHz for detection pulses; Siemens Medical Solutions, Mountain View, California, USA). The detection pulses measured the shear wave velocity (SWV), which was considered to directly relate to tissue stiffness.

All ARFI stiffness measurements were performed by the same hepatologist, who was experienced in digestive system ultrasonography and blinded to the patient data. The right lobe [17] of the liver (Couinaud segments VIII, V, VII, VI) was approached intercostally [18], with the patient lying in a dorsal decubitus position [12] with both arms above the head and holding their breath during virtual touch quantification (VTQ) measurements. During each measurement, the region of interest (ROI) (10x5 mm) was placed at the middle of the acoustic field, avoiding large blood vessels and biliary tracts.

SWV results were recorded in meters per second (m/s) and stored as jpg image files. Each patient received 10 successful LSMs (failed measurements were defined as SWV = "x.xx m/s") [40]. Reliable cases were defined as those with an interquartile range (IQR) of less than 30% of the median of 10 successful LSMs, and a successful rate of LSMs greater than 60%. Other cases were deemed unreliable and excluded [9,10,19].

### Histology

Senior hepatologists performed the percutaneous right lobe liver biopsy. All biopsy specimens were interpreted by an expert pathologist blinded to the results of LSMs and patient data. Biopsy specimens at least 15 mm in length containing at least five portal tracts were defined adequate [6]. Liver fibrosis and necroinflammatory activity were staged and graded using the METAVIR scoring system: F0, no fibrosis; F1, portal fibrosis without septa; F2, portal fibrosis with a few septa; F3, numerous septa without cirrhosis; and F4, cirrhosis; A0, none; A1, mild; A2, moderate; and A3, severe necroinflammatory activity [41]. Steatosis categories were S0, no steatosis; S1, 1 to 5% (percentage of hepatocytes containing visible macrovesicular steatosis); S2, 6 to 32%; S3, 33 to 66%; and S4, 67 to 100% [42].

### Statistical analysis

The intraobserver correlations of ARFI LSM were evaluated using an intraclass correlation coefficient (ICC) for independent LSMs performed on two separate occasions in 20 patients by the same hepatologist with experience in 50 training cases.

Spearman’s rank correlation was used to evaluate the significance of correlations between continuous variables and liver fibrosis stages. Intergroup differences were analyzed using Fisher’s exact test for proportions, and the Student’s *t*-test or one-way ANOVA for continuous variables, when appropriate.

Receiver operating characteristic (ROC) curves were used to optimize the cutoff values [43] and evaluate diagnostic performance.

After univariate linear regression, variables with a *P* value of less than .25 were included in the subsequent stepwise multiple linear regression modelling. In addition to the two potential confounders of age and sex, the three final multiple linear regressions included variables maximizing the adjusted R^2^ in each stepwise regression to identify significant independent explanatory factors for LSM. Four final multiple linear regression models were used to delineate the necroinflammatory effects on LSM after adjusting for serum ALT levels (model estimates are reported in the discussion section), ALT/upper limit of normal (ULN, 40 IU/L) categories (model 1), ActiTest A scores (model 2) and METAVIR A grades (model 3). Binary logistic regression was also used to examine the necroinflammatory effects on false positivity in liver fibrosis evaluation using ARFI LSM in patients with METAVIR F1, F2 and F3.

Data were analyzed using SPSS version 17.0 for Microsoft Windows (SPSS, Chicago, Illinois, USA). A two-sided *P* value of < .05 indicated statistical significance.

## Results

### Patients

Among the 137 patients, this study excluded 2 patients with liver cancer (both HCC), 2 with ALD, 1 with end stage renal disease, 2 with unreliable LSM results, and 3 with inadequate specimen quality. Thus, 127 patients entered the complete analysis.

**Figure 1:**
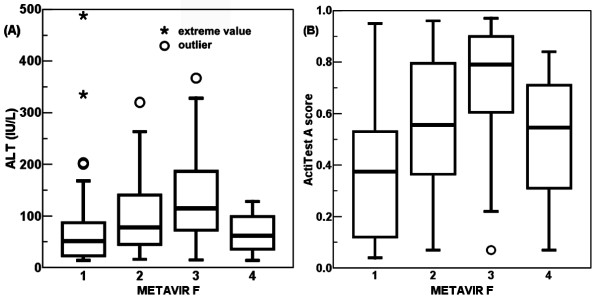
**Box plot of necroinflammatory degrees corresponding to the METAVIR fibrosis (F) stages.** The Spearman’s rank correlation coefficients were 0.196 (*P* = .027) between serum alanine aminotransferase (ALT) levels and METAVIR F stages (**A**); 0.305 (*P* < .001) between ActiTest A scores and METAVIR F stages (**B**). Pair-wise comparisons of ActiTest A scores showed significant differences between groups F1, F2, and F3: all *P* < .005.

**Figure 2:**
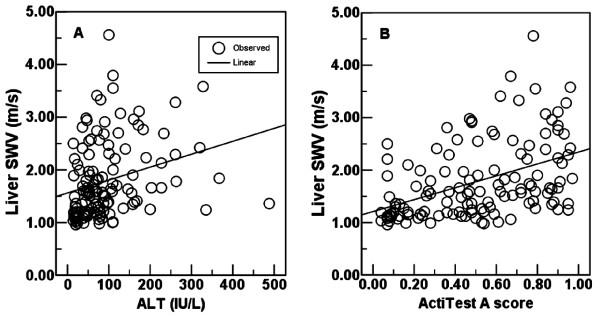
**Univariate linear regressions between necroinflammatory degrees and liver shear wave velocity (SWV).** Univariate linear regressions were both significant between serum alanine aminotransferase (ALT) levels and liver SWV (R^2^ = 0.074, *P* = .002) (**A**), and between ActiTest A scores and liver SWV (R^2^ = 0.198, *P* < .001) (**B**).

Age, serum ALT, total bilirubin, HCV RNA, international normalized ratio (INR) of prothrombin time, platelet count, liver SWV, and F score of FibroTest differed significantly between the non-cirrhotic and cirrhotic groups (Table 1). The serum ALT levels ranged from 14 to 488 IU/L, and Actitest A scores ranged from 0.04 to 0.97. Spearman’s rank correlation coefficients were 0.196 (*P* = .027) between ALT and METAVIR F stages; 0.305 (*P* < .001) between ActiTest A scores and METAVIR F stages (Figure 1). Univariate linear regressions were both significant between ALT and ARFI LSM (R^2^ = 0.074, *P* = .002), and between ActiTest A scores and ARFI LSM (R^2^ = 0.198, *P* < .001) (Figure 2).

**Table 1:** Patient characteristics

**Variable**	**METAVIR F0-3**	**METAVIR F4**	***P*value**
	**n = 109**	**n = 18**	
Age, year	51.6(1.2)	62.7(1.5)	<.001
Gender (n)			1.000
Male	51	8	
Female	58	10	
BMI, kg/m^2^	24.64(0.33)	24.02(0.79)	.477
HCV genotype (n)			1.000
1	53	9	
Non-1	56	9	
HCV RNA, x 10^6^, copies/mL	8.16(1.21)	1.02(0.52)	<.001
ALT, IU/L	97.94(8.24)	64.28(8.07)	.005
ALT/ULN (n)			.273
<1x	26	5	
≧1x <2x	35	7	
≧2x <3x	21	5	
≧3x <4x	7	1	
≧4x	20	0	
ActiTest, A score	0.51(0.03)	0.51(0.06)	.928
Bilirubin, umol/L	16.80(0.54)	22.36(2.00)	<.001
Cr, umol/L	71.80(4.34)	65.42(4.35)	.301
INR	1.02(0.01)	1.12(0.03)	<.001
Na, meq/L	137.90(0.24)	137.83(0.60)	.918
Platelet, x 10^9^/L	176.61(5.67)	121.44(9.73)	<.001
Child-Pugh grade (n)			
A		16	
B		2	
C			
METAVIR F (n)			
F0	0		
F1	46		
F2	40		
F3	23		
F4		18	
METAVIR A (n)			.484
A0	31	3	
A1	60	10	
A2	16	5	
A3	2	0	
Hepatic steatosis (n)			1.000
S0	15	2	
S1	37	6	
S2	52	10	
S3	4	0	
S4	1	0	
Liver SWV, m/s	1.65(0.06)	2.62(0.19)	<.001
FibroTest, F score	0.57(0.03)	0.79(0.04)	<.001

Continuous variables were presented as mean (standard error) and examined using the Student’s *t* test. Categorical variables were estimated using Fisher’s exact test. ALT, serum alanine aminotransferase; Cr, serum creatinine; INR, international normalized ratio of prothrombin time; METAVIR A, activity; METAVIR F, fibrosis; Na, serum sodium; SWV, shear wave velocity.

### Histology

The mean length of the included liver biopsy specimens was 21.7 ± 3.3 mm (standard deviation, SD) (range, 15 to 32 mm). Using the METAVIR F scoring system, 46 patients were staged as F1, 40 as F2, 23 as F3, and 18 as F4.

### ARFI LSM

**Figure 3:**
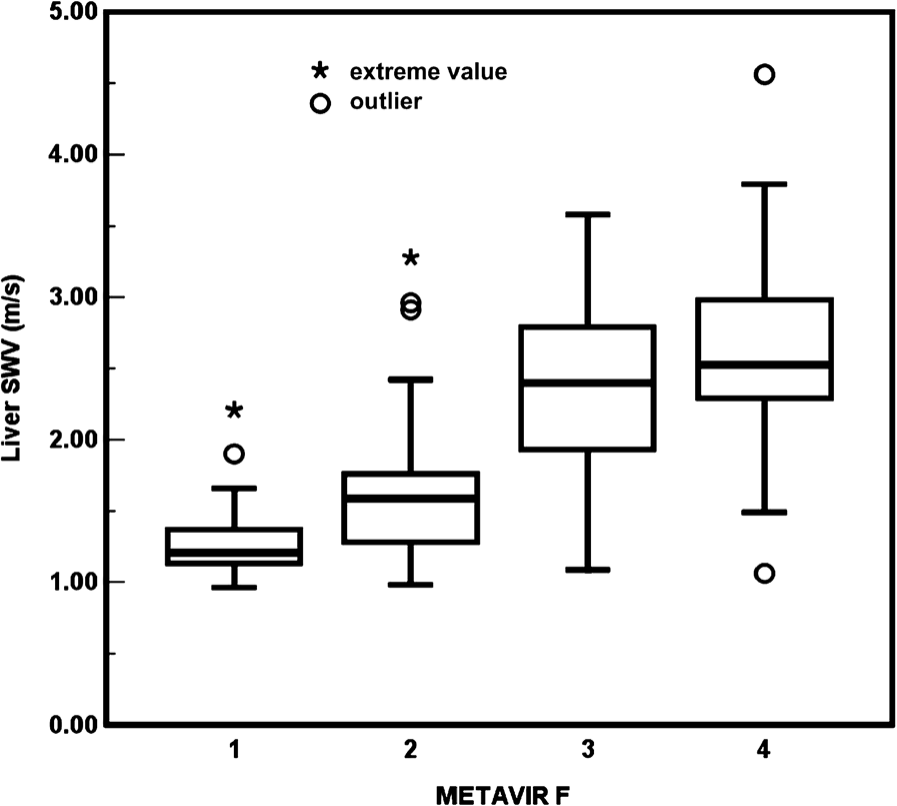
**Box plot of liver stiffness corresponding to the METAVIR fibrosis (F) stages.** The overall inter-group difference of liver shear wave velocity (SWV) among METAVIR F1 to F4 was significant (*P* < .001). Pair-wise comparisons also showed significant differences between groups: all *P* < .001, except *P* = .412 for F3 versus F4.

The ICC was 0.993 (95% CI, 0.981-0.997; *P* < .001) for LSM. The descriptive statistics of the liver SWVs for the METAVIR stages were as follows: F1: mean, 1.283 ± 0.037 m/s (standard error of mean, SE); median, 1.205 m/s (range, 0.96 to 2.21); F2: mean, 1.630 ± 0.081 m/s; median, 1.590 m/s (range, 0.98 to 3.28); F3: mean, 2.433 ± 0.134 m/s; median, 2.395 m/s (range, 1.09 to 3.58); F4: mean, 2.619 ± 0.187 m/s; median, 2.525 m/s (range, 1.06 to 4.56) (Figure 3).

The Spearman’s rank correlation coefficient between liver SWV and METAVIR F stages was 0.696 (*P* < .001). The overall intergroup difference in liver SWVs between METAVIR F1 to F4 was significant (*P* < .001). Pair-wise comparisons also showed significant differences between groups: all *P* < .001, except *P* = .412 for F3 versus F4.

**Figure 4:**
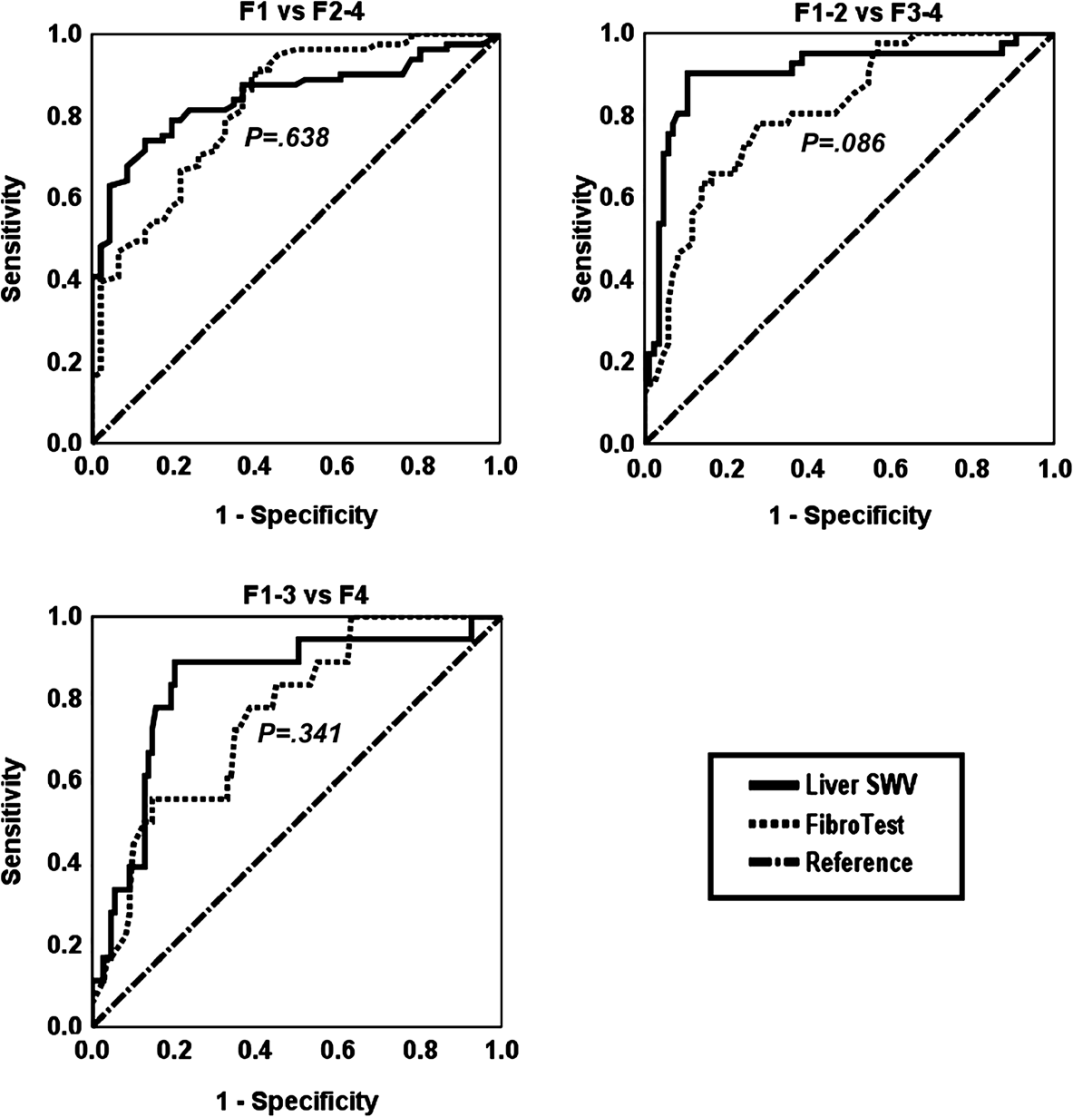
**Receiver operating characteristic (ROC) curves to classify the METAVIR fibrosis stages.** Two diagnostic modalities, liver shear wave velocity (SWV), and FibroTest (F score) were compared. *P* value: significance for comparisons of areas under ROC curves between using liver SWV and FibroTest.

To classify METAVIR F stages, the areas under the ROC curves (AUCs) were ARFI LSM, 0.847 (95% CI, 0.779-0.914) and FibroTest, 0.823 (95% CI, 0.748-0.898), for F1 versus F2-4; ARFI LSM, 0.902 (95% CI, 0.835-0.970) and FibroTest, 0.812 (95% CI, 0.735-0.888), for F1-2 versus F3-4; ARFI LSM, 0.831 (95% CI, 0.723-0.939) and FibroTest, 0.757 (95% CI, 0.648-0.865), for F1-3 versus F4. A comparison of the AUCs [16] using liver SWV and FibroTest results showed insignificant differences between the following groups: F1 versus F2-4, *P* = .638; F1-2 versus F3-4, *P* = .086; F1-3 versus F4, *P* = .341 (Figure 4).

The optimal cutoff values to classify METAVIR F stages using ARFI LSM were 1.55 m/s for F1 versus F2-4 (74.1% sensitivity, 87.0% specificity, 90.9% positive predictive value (PPV), 65.6% negative predictive value (NPV), 78.8% concordance, 21.2% discordance), 1.81 m/s for F1-2 versus F3-4 (90.2% sensitivity, 89.5% specificity, 80.4% PPV, 95.0% NPV, 89.7% concordance, 10.3% discordance), and 1.98 m/s for F1-3 versus F4 (88.9% sensitivity, 79.8% specificity, 42.1% PPV, 97.8% NPV, 81.1% concordance, 18.9% discordance) (Table 2).

**Table 2:** The diagnostic performance of liver SWV and FibroTest in classifying METAVIR fibrosis (F) stages

	**METAVIR**		
	**F1*vs*F2-4**	**F1-2*vs*F3-4**	**F1-3*vs*F4**
	***Liver SWV***		
AUC (95% CI)	0.847(0.779-0.914)	0.902 (0.835-0.970)	0.831 (0.723-0.939)
SE of AUC	0.034	0.035	0.055
Cutoff (m/s)	1.55	1.81	1.98
Sensitivity	74.1%	90.2%	88.9%
Specificity	87.0%	89.5%	79.8%
PPV	90.9%	80.4%	42.1%
NPV	65.6%	95.0%	97.8%
Concordance	78.8%	89.7%	81.1%
Discordance	21.2%	10.3%	18.9%
+LR	5.7	8.6	4.4
-LR	0.3	0.1	0.1
DOR(95% CI)	19.0(7.1-51.3)	79.1(22.9-273.8)	31.6(6.8-147.9)
	***FibroTest***		
AUC (95% CI)	0.823(0.748-0.898)	0.812 (0.735-0.888)	0.757 (0.648-0.865)
SE of AUC	0.038	0.039	0.055

Concordance = (true positive n + true negative n)/127, and discordance = (false positive n + false negative n)/127; AUC, area under the receiver operating characteristic curve; DOR, diagnostic odds ratio; LR, likelihood ratio; NPV, negative predictive value; PPV, positive predictive value; SE, standard error; SWV, shear wave velocity.

### Statistical analysis

After excluding the HCV genotype (*P* = .810), serum creatinine (Cr) (*P* = .997), and sodium (Na) (*P* = .388), univariate linear regression modelling selected BMI, HCV RNA, METAVIR F stages, ALT/ULN (40 IU/L) categories (model 1), ActiTest A scores (model 2) and METAVIR A grades (model 3), hepatic steatosis categories, bilirubin, INR, and platelet count (all *P* < .25), in addition to age (*P* = .001) and sex (*P* = .425), for the subsequent stepwise multiple linear regressions. The HCV RNA, hepatic steatosis categories, and serum bilirubin were excluded from the stepwise regressions for all three models.

Finally, in addition to BMI, METAVIR F stages, and platelet count, multiple linear regression models 1, 2, and 3 consistently identified serum ALT/ULN categories (model 1), ActiTest A scores (model 2) and METAVIR A grades (model 3) as significant independent explanatory factors for LSM (Table 3).

**Table 3:** Three multiple linear regression models to identify independent significant factors that explain liver stiffness

**Variable**	**Model 1**			**Model 2**			**Model 3**		
	**B**	**SE**	***P***	**B**	**SE**	***P***	**B**	**SE**	***P***
Age, year	-.003	.004	.512	-.004	.004	.353	-.004	.004	.366
Male gender	-.193	.084	**.024**	-.194	.083	**.020**	-.124	.085	.147
BMI, kg/m^2^	.032	.013	**.013**	.036	.012	**.005**	.026	.012	**.041**
INR	.972	.572	.092	.890	.564	.117	1.146	.573	**.048**
Platelet, x10^9^/L	-.003	.001	**.002**	-.003	.001	**.003**	-.002	.001	**.007**
METAVIR									
F2	.089	.113	.430	.049	.113	.666	.123	.114	.284
F3	.739	.154	**<.001**	.670	.155	**<.001**	.670	.161	**<.001**
F4	.989	.168	**<.001**	.954	.165	**<.001**	.892	.171	**<.001**
**ALT/ULN**									
**≧1<2x**	.307	.110	**.006**						
**≧2<3x**	.429	.122	**.001**						
**≧3**	.523	.133	**<.001**						
**ActiTest A score**				.717	.163	**<.001**			
**METAVIR A**									
**A1**							.189	.100	.061
**A2-3**							.551	.144	**<.001**
R^2^		.661			.662			.651	
adjusted R^2^		.629			.636			.620	

Variable references: female gender, METAVIR F1, ALT/ULN <1x, METAVIR A0; ALT, serum alanine aminotransferase; B, coefficient; INR, international normalized ratio of prothrombin time; SE, standard error of coefficient; ULN, upper limit of normal.

Thirty-two patients out of the 109 patients with METAVIR F1, F2 and F3 were false positive (32/109, 29%). False positive case numbers were as follows: 8 (8/46, 17%) (ARFI LSM > 1.55 m/s) in METAVIR F1; 8 (8/40, 25%) (LSM > 1.81 m/s) in F2; 16 (16/23, 70%) (LSM > 1.98 m/s) in F3 stages.

**Figure 5:**
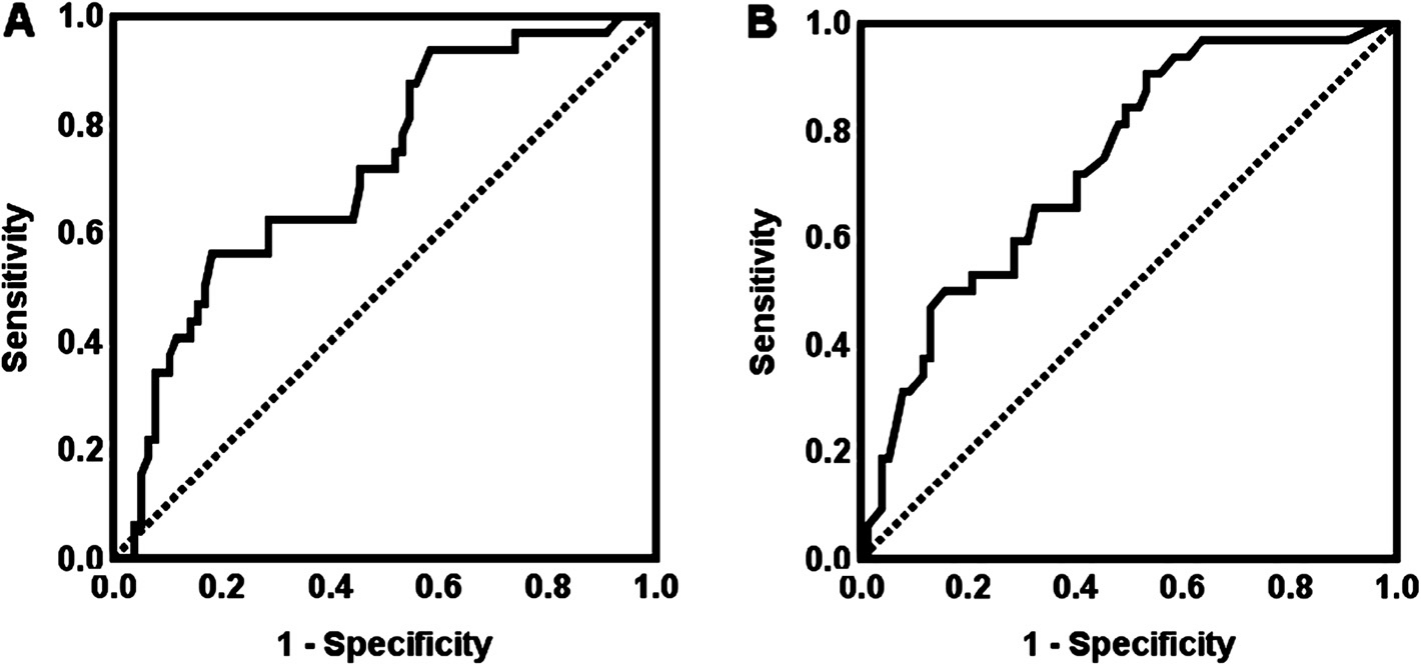
**Receiver operating characteristic (ROC) curves to classify the false positivity in METAVIR F1 to F3.** Using alanine aminotransferase (ALT) levels (**A**) to discriminate the false positive in liver fibrosis evaluation using acoustic radiation force impulse elastography (n = 32) from the non false positive (n = 77) of the 109 patients with METAVIR F1, F2 and F3, the area under ROC curve (AUC) was 0.715 (standard error, SE, 0.053; 95% CI, 0.612-0.819; *P* < .001). The ALT level of 109.5 IU/L was the optimal cutoff value with a sensitivity of 56.3% and a specificity of 81.8%. Using the ActiTest A scores (**B**), the optimal cutoff was 0.35 (AUC, 0.736; SE, 0.051; 95% CI, 0.636-0.835; *P* < .001; sensitivity, 93.8%; specificity, 41.6%).

Using ALT levels to discriminate the false positive (n = 32) from the non false positive (n = 77) of the 109 patients with METAVIR F1, F2 and F3, the AUC was 0.715 (SE, 0.053; 95% CI, 0.612-0.819; *P* < .001). The ALT level of 109.5 IU/L was the optimal cutoff value with a sensitivity of 56.3% and a specificity of 81.8%. Using the ActiTest A scores, the optimal cutoff was 0.35 (AUC, 0.736; SE, 0.051; 95% CI, 0.636-0.835; *P* < .001; sensitivity, 93.8%; specificity, 41.6%)(Figure 5).

Using “ALT/ULN <1x”, “METAVIR A0”, and “ActiTest A score 0.00-0.35” as reference categories, univariate binary logistic regressions showed significant necroinflammatory effects on the false positivity of LSM to stage fibrosis in the 32 patients with METAVIR F1, F2 and F3. “ALT/ULN ≧3x” had an odds ratio (OR) of 15.0 (95% CI, 2.9-76.6; *P* = .001). “METAVIR A2-3”: OR, 32.7; 95% CI; 6.4-166.5; *P* < .001. “ActiTest A score 0.36-0.75”: OR, 8.4; 95% CI, 1.7-40.8; *P* = .002. “ActiTest A score 0.76-1.00”: OR, 11.9; 95% CI, 2.5-57.2; *P* = .002 (Table 4).

**Table 4:** Factors associated with false positivity in patients with METAVIR F1, F2 and F3

**Variable**	**Non FP**	**FP**	**OR(95% CI)**	***P*value**
	**n = 77**	**n = 32**		
Age, year	50.8(1.4)	53.4(2.0)		.316
Gender, male/female (n)	35/42	16/16		.679
BMI, kg/m^2^	24.34(0.37)	25.37(0.68)		.155
HCV genotype, 1/non-1 (n)	38/39	15/17		.836
HCV RNA, x 10^6^, copies/mL	7.14(1.12)	10.61(3.12)		.194
ALT, IU/L	83.01(9.40)	133.88(15.02)		.004
ALT/ULN (n)				
<1x	24	2	reference	
≧1x <2x	25	10	4.8(0.9-24.2)	.057
≧2x <3x	16	5	3.8(0.6-21.7)	.140
≧3x	12	15	15.0(2.9-76.6)	.001*
ActiTest A score (n)				.
0.00-0.35	31	2	reference	
0.36-0.75	24	13	8.4(1.7-40.8)	.008*
0.76-1.00	22	17	11.9(2.5-57.2)	.002*
Bilirubin, umol/L	15.76(0.56)	19.30(1.12)		.002
Cr, umol/L	70.82(2.82)	74.14(4.55)		.817
INR	1.00(0.01)	1.05(0.02)		.007
Na, meq/L	137.94(0.28)	137.81(0.46)		.527
Platelet, x 10^9^/L	184.21(6.20)	158.31(9.37)		.025
METAVIR F1/2/3 (n)	38/32/7	8/8/16		<.001
METAVIR A (n)				
A0	28	3	reference	
A1	45	15	3.1(0.8-11.7)	.094
A2-3	4	14	32.7(6.4-166.5)	<.001*
Hepatic steatosis S0/1/2/3/4(n)	10/28/35/3/1	5/9/17/1/0		.903
Liver SWV, m/s	1.34(0.03)	2.40(0.11)		<.001
FibroTest, F score	0.51(0.03)	0.72(0.04)		<.001

Thirty-two patients of the 109 patients with METAVIR F1, F2 and F3 were false positive (32/109, 29%) in liver fibrosis evaluation using acoustic radiation force impulse elastography. False positive case numbers were: 8 (8/46, 17%) (ARFI LSM > 1.55 m/s) in METAVIR F1; 8 (8/40, 25%) (LSM > 1.81 m/s) in F2; 16 (16/23, 70%) (LSM > 1.98 m/s) in F3 stages. Continuous variables were presented as mean (standard error) and examined using the Student’s *t*-test. Categorical variables were estimated using Fisher's exact test or univariate binary logistic regression*. ALT, serum alanine aminotransferase; ARFI, acoustic radiation force impulse; CI, confidence interval; Cr, serum creatinine; FP, false positivity; INR, international normalized ratio of prothrombin time; LSM, liver stiffness measurement; METAVIR A, activity; METAVIR F, fibrosis; Na, serum sodium; non FP, true positive + true negative + false negative; OR, odds ratio; SWV, shear wave velocity.

## Discussion

In the chronically injured liver, fibrogenesis is the complex dynamic interplay among various hepatic cell types and mediators in which the process of perpetuation follows initiation [4]. With the clinical application of magnetic resonance (MR) and ultrasound-based LSM, studies using MR elastography (MRE) [44], FibroScan, and ARFI elastography have demonstrated significant correlations between liver stiffness and liver fibrosis. However, liver stiffness and liver fibrosis are not equivalent.

In addition to the accumulation of the fibrotic extracellular matrix, other components of chronic liver disease, including cholestasis [31,32], cardiac congestion [33], and, particularly, the degree of necroinflammatory activity, can exaggerate ultrasound-based LSM. Patients with CHC usually manifest relatively stable serum ALT levels compared with the abrupt and fluctuating ALT levels in patients with chronic hepatitis B (CHB). Swelling of hepatocytes, interstitial edema, and infiltrates of inflammatory cells may increase liver stiffness in patients with acute hepatitis [45]. After adjusting for other demographic and biological covariates, the results of our study indicate that a 100 IU/L increase in serum ALT levels augmented liver stiffness values by approximately 0.155 m/s (model R^2^ = 0.630, adjusted R^2^ = 0.602).

ActiTest is a biomarker of liver necroinflammatory histological activity validated in patients with CHC. The accuracy of ActiTest for grading necroinflammatory activity in HCV-infected patients was significantly higher than serum ALT alone [34]. In our study, the ActiTest A score model (R^2^ =0.662, adjusted R^2^ = 0.636) was superior to the models using serum ALT levels (R^2^ = 0.630, adjusted R^2^ = 0.602), ALT/ULN categories (R^2^ =0.661, adjusted R^2^ = 0.629) and METAVIR A grades (R^2^ = 0.651, adjusted R^2^ = 0.620) in explaining the LSM results.

The cutoffs of ARFI LSM in this study (1.55 m/s for F1 versus F2-4; 1.81 m/s for F1-2 versus F3-4) were in part higher than those in the latest study on 139 CHC cases by Rizzo et al. (1.3 m/s for F1 versus F2-4; 1.7 m/s for F1-2 versus F3-4)[12]. Necroinflammatory effects explain most of the differences. First, the overall necroinflammatory degree was higher (Student’s *t*-test, *P* = .034) in this study (mean ALT, 93.2; SE, 7.2 IU/L; n = 127) than for Rizzo et al. (mean ALT, 77.2; SD, 33.0 IU/L; n = 139). Second, the distribution of necroinflammation tended to be more severe in METAVIR F3 and F2 than F1 stages in this study (Figure 1). Thus, the LSM values were more augmented in F3 and F2 than F1 stages. The analyses of false positivity in patients with F1 to F3 also correlated well with the necroinflammatory effects (Table 4). Third, cirrhotic Taiwanese patients (mean age, 62.7; SE, 1.5 years; n = 18) referred to the unit for SOC tended to be older (Student’s *t*-test, *P* = .0083) than those in the study by Rizzo et al. (mean age, 55; SD, 12 years; n = 139) [12]. Despite the advanced countrywide education and health insurance coverage, these patients were characterized by poor patient compliance and late diagnosis. Although age did not affect LSM significantly, as demonstrated in models in the present and previous studies, an older age may have resulted in significant fibrosis progression in these cirrhotic Taiwanese patients despite the relatively compensated liver reserves.

Despite the variation among previous investigations, several recent studies have demonstrated the necroinflammatory effects on liver stiffness by evolving analyses.

In a longitudinal study using FibroScan, Sagir et al. [21] observed false positivity for cirrhosis (cutoff > 12.5 kPa) in 15 of 20 non-cirrhotic patients with acute liver damage of various etiologies. In 6 patients, the LSMs dropped below 12.5 kPa with normalized ALT levels during follow-up. Using a longitudinal analysis, Arena et al. [20] demonstrated significant correlations between sequential serum ALT levels and LSM results at different time points. Although these studies showed the need for caution when analyzing LSM in patients with necroinflammatory flares, they did not include regression estimates. Seo et al. [25] demonstrated that peak ALT levels significantly explained peak LSMs in 31 patients in acute hepatitis A via linear regressions adjusting for age and sex.

A cross-sectional study by Le et al. [23] showed that LSM using FibroScan in 158 patients was independently associated with histological necroinflammatory grading, but irrespective of serum ALT levels. Fung et al. [26] reported a suboptimal PPV (as low as 10%) for LSM using FibroScan to diagnose true cirrhosis in 102 patients (median age, 41; range, 18–63 years) with active hepatitis B (median serum ALT, 89; range, 46–501 IU/L). Multiple logistic regressions by Myers et al. [29] showed that serum ALT levels greater than the optimal cutoff value 60 IU/L from ROC analysis were significantly correlated with the discordance (at least 2 stages between FibroScan and biopsy). Chan et al. [24] and Kim et al. [27] proposed distinct sets of cutoff values stratified by distinct ALT profiles.

Tapper et al. [30] further delineated the positive necroinflammatory effects on LSM using FibroScan through linear regressions in 684 HCV patients with METAVIR F0, F1 and F2. Logistic regressions also showed that false positivity of liver fibrosis staging was associated with both histological and serum hepatic necroinflammatory activity. Using 14.5 kPa as the cutoff of cirrhosis, grade 3 inflammation had an OR of 9.10 (95% CI, 2.49-33.4). Likewise, serum levels of ALT greater than 80 IU/L and 120 IU/L had ORs of 3.84 (95% CI, 2.10-7.00) and 4.10 (95% CI, 2.18-7.69) over references, respectively. Yoon et al. [15] used ARFI elastography to demonstrate a significant correlation (Pearson's r = 0.431, *P* < .05) between LSM values and serum ALT levels, and a marked positive effect of histological necroinflammatory activity on LSM. However, this study did not adjust for other relevant essential covariates. ARFI elastography performed potentially better for patients with normal ALT than for those with high ALT (AUCs, 0.88 versus 0.73 for METAVIR F1-2 versus F3-4, 0.92 versus 0.72 for F1-3 versus F4). However, AUCs were not statistically compared. The cutoff values tended to be lower for patients with normal ALT than for those with high ALT levels (ALT > 40 IU/L)(1.09 versus 1.16 m/s for F1-2 versus F3-4, and 1.81 versus 2.23 m/s for F1-3 versus F4, respectively).

In contrast to results indicating positive necroinflammatory effects on liver stiffness, Harata et al. [32] identified a negative correlation between serum ALT levels and liver stiffness in patients with cholestasis. Cholestasis is a condition in which the release of hydrostatic pressure with synchronous necroinflammatory activity can, paradoxically, reduce the values of LSM using FibroScan. Rizzo et al. [12] found distinct necroinflammatory effects on LSM between using FibroScan and ARFI elastography. Colombo et al. [13] demonstrated an insignificant (Spearman’s rank) correlation between necroinflammation and LSM. The serum ALT specific cutoffs of 202 CHB patients in a study by Cardoso et al. did not increase the diagnostic performances using FibroScan for liver fibrosis evaluation [28].

Although hepatic steatosis is prevalent in patients with CHC, the linear regression analysis in this study did not identify steatosis as a significant independent explanatory factor for the ARFI LSM results. However, larger sample sizes are required to further delineate the effects of more severe forms of steatosis (S3, S4) on ARFI LSM results. Motosugi et al. [46] also demonstrated the insignificance of different ARFI LSM results among four grades of steatosis (*P* = .9018). Using MRE in a mouse model, Yin et al. [47] showed that steatosis did not correlate significantly with liver stiffness at each liver fibrosis stage.

This study shows that BMI significantly and independently explain the results of ARFI LSM (Table 3). Similarly, Roulot et al. [48] used FibroScan to show that liver stiffness was significantly higher (*P* = .0005) in obese patients (with BMI > 30 kg/m^2^) than in overweight or normal patients, after adjusting for age, sex, ALT, aspartate aminotransferase (AST) and ferritin. Baba et al. also demonstrated a significant association between BMI and liver stiffness using FibroScan. Hepatic steatosis, however, was not evaluated in adjusted models [49]. Conversely, Talwalkar et al. [50] reported an insignificant (linear) correlation between BMI and LSM results using MRE.

Similar to the report by Talwalkar et al. [50], the effects of age and sex on ARFI LSM were insignificant in this study. Previously demonstrated as a significant factor explaining liver fibrosis evaluation [51], platelet count was strongly associated with ARFI LSM after adjustment, unlike the inconsistency and insignificance of INR (Table 3).

To minimize histology bias [6,52], the percutaneous liver biopsy in this study immediately followed ARFI LSM. Specimens were of adequate length, and an expert pathologist interpreted histological findings. Unreliable cases or failed ARFI measurements were primarily derived from obese patients with poor transient apnea maneuvers. Thus, future studies may analyze factors associated with unreliable cases or failure measurements. The present analyses show that the R^2^ values were modest for the regression models; therefore, other potential explanatory factors, especially direct tissue markers [45], must be identified to construct an optimal explanatory model (Table 3).

Future analyses would require a larger sample size to develop cutoffs and perform validations that are potentially more reliable and stable than those of this study. These investigations may compare the diagnostic performances of ARFI LSM with another promising diagnostic modality: MRE. Although FibroTest was initially proposed as a similar first-line approach to histology for prediction of 5-year survival in patients with CHC [37,38], it may also serve as a standard of reference, in addition to METAVIR F staging, for evaluating the diagnostic value of ARFI LSM in baseline or chronological analysis.

The limitations of this study include the lack of stratification of patients with cirrhosis into compensated and decompensated (excluded) groups. The absence of a decompensated group, which may have a high and broad spectrum for LSM, may have caused the insignificant differences in LSM results between METAVIR F3 and F4 (*P* = .412) before adjusting for other covariates. Further analyses should identify optimal cutoffs for LSM to stratify the broad cirrhosis category and enable risk estimation of end points in chronological analyses [53]. This study does not identify the cutoff for LSM between METAVIR F0 and F1-F4 because of the lack of well-established criteria for recruiting true cases with F0 fibrosis without liver biopsy.

Future noninvasive liver fibrosis evaluation tools should focus on exact staging of fibrosis, rather than classifying patients into categories of milder versus more severe fibrosis stages. In the milder strata of liver fibrosis, necroinflammation can easily overwhelm the fibrotic effects on LSM [30]. In this study, the diagnostic performance of ARFI LSM was potentially limited when distinguishing between METAVIR F1 and F2 categories too. The insignificant regression coefficient in Table 3 reflects the potentially limited sensitivity for ARFI LSM in distinguishing F2 from F1. Therefore, further research is needed prior to the clinical application of ARFI LSM for surveillance of progression, or regression, of liver fibrosis. The real clinical relevance between METAVIR F1 and F2 is still not known with respect to either prognosis or risk of disease progression over the short to intermediate term of 5 to 10 years [5]. Future studies should develop algorithms based on a combination of ARFI LSM and essential serum markers for liver fibrosis evaluation to minimize false positivity of fibrosis staging [24,27,54]. A further limitation is that this study did not perform standardization of AUCs based on the prevalence of liver fibrosis stages [55].

## Conclusions

In conclusion, the degree of concurrent hepatic necroinflammatory activity independently and significantly exaggerated liver fibrosis evaluation using ARFI LSM. However, comparisons of concurrent FibroTest and ARFI LSM indicate that ARFI LSM may provide a promising alternative, or adjunctive predictive solution, for evaluating liver fibrosis in patients with CHC.

## Abbreviations

ALD: Alcoholic Liver Disease; ALT: ALanine aminoTransferase; AST: ASpartate aminoTransferase; ARFI: Acoustic Radiation Force Impulse; AUC: Area Under the receiver operating characteristic Curve; BMI: Body Mass Index; CHB: Chronic Hepatitis B; CHC: Chronic Hepatitis C; CI: Confidence Interval; Cr: Creatinine; DOR: Diagnostic Odds Ratio; FP: False Positive; GGT: γ-Glutamyl Transpeptidase; HBV: Hepatitis B Virus; HCV: Hepatitis C Virus; HCC: HepatoCellular Carcinoma; HIV: Human Immunodeficiency Virus; ICC: Intraclass Correlation Coefficient; INR: International Normalized Ratio; IQR: InterQuartile Range; +LR: Positive Likelihood Ratio; -LR: Negative Likelihood Ratio; LSM: Liver Stiffness Measurement; METAVIR A: Activity; METAVIR F: Fibrosis; MRE: Magnetic Resonance Elastography; Na: Sodium; NPV: Negative Predictive Value; PPV: Positive Predictive Value; ROC: Receiver Operating Characteristic; ROI: Region Of Interest; SD: Standard Deviation; SE: Standard Error of mean; SOC: Standard Of Care; SWV: Shear Wave Velocity; ULN: Upper Limit of Normal; VTQ: Virtual Touch Quantification.

## Competing interests

The authors declare that they have no competing interests.

## Authors’ contributions

SHC, YFL and CYP designed the study and analyzed the data. SHC performed the ARFI LSMs. IPC performed the histological interpretations. SHC, YFL, HCL, JTK, CYP, PHC, WPS, and IPC participated in the drafting of the manuscript. All of the authors read and approved the final manuscript.

## Pre-publication history

The pre-publication history for this paper can be accessed here:

http://www.biomedcentral.com/1471-230X/12/105/prepub
